# Mobile, Collaborative Situated Knowledge Creation for Urban Planning

**DOI:** 10.3390/s120506218

**Published:** 2012-05-10

**Authors:** Gustavo Zurita, Nelson Baloian

**Affiliations:** 1 Management and Information System Department, Universidad de Chile, Diagonal Paraguay 257, Santiago, Chile; E-Mail: gzurita@fen.uchile.cl; 2 Department of Computer Sciences, Universidad de Chile, Blanco Encalada 2120, Santiago, Chile

**Keywords:** geo-collaboration, Knowledge Creation, mobile collaborative work

## Abstract

Geo-collaboration is an emerging research area in computer sciences studying the way spatial, geographically referenced information and communication technologies can support collaborative activities. Scenarios in which information associated to its physical location are of paramount importance are often referred as Situated Knowledge Creation scenarios. To date there are few computer systems supporting knowledge creation that explicitly incorporate physical context as part of the knowledge being managed in mobile face-to-face scenarios. This work presents a collaborative software application supporting visually-geo-referenced knowledge creation in mobile working scenarios while the users are interacting face-to-face. The system allows to manage data information associated to specific physical locations for knowledge creation processes in the field, such as urban planning, identifying specific physical locations, territorial management, *etc.*; using Tablet-PCs and GPS in order to geo-reference data and information. It presents a model for developing mobile applications supporting situated knowledge creation in the field, introducing the requirements for such an application and the functionalities it should have in order to fulfill them. The paper also presents the results of utility and usability evaluations.

## 1. Introduction

Urban planning is the label adopted by a broad research agenda addressing the interaction between information technology and planning, including various key concerns such as territorial management, policy making, governance, citizenship and participation [1]. The main vision driving the software application design was supplying various stakeholders—architects, urban designers, city planners, and public administrators—with a collaborative software application supporting the creation of new perceptions and ideas regarding city planning.

The integration of geo-referenced data and information with decision models is not new. Actually, it has led to an emerging category of Geographic Information Systems known as Collaborative Spatial Decision Making [2,3]. A Collaborative Spatial Decision Making system usually provides the following functionalities: collecting geo-referenced data and information, identifying locations according to a set of criteria, generating a brainstorming session, displaying and analyzing data, and decision making support.

The integration of visual geo-referenced data and information with a knowledge creation model applied to design a concrete software application supporting face-to-face social interaction among users in mobile working scenarios has not been proposed in the literature so far. Various authors mention the relevance of physical context and situated knowledge for: (a) improving knowledge transfer based on interaction with the real physical context while sharing explicit knowledge during face-to-face interactions among users and experts [4]; (b) facilitating knowledge extraction from information associated to physical context or locations [2,5]; and (c) supporting knowledge acquisition among users located in various places while working in a virtually co-located workspace. In spite of the reasons already explained, just few research works combine the use of geo-referenced information with knowledge management, which falls short from knowledge creation. For example, Convertino, Ganoe, *et al.* [6] investigated strategies supporting knowledge sharing in distributed, synchronous collaboration, using a multiple view approach to support common ground in geo-collaboration; Gahegan and Pike [7] developed a situated knowledge representation of geographical information based on a novel approach to conceive, construct and compare the concepts developed and used by geographers, environmental scientists and other earth science researchers to help describe, analyze and ultimately understand their subject of study.

It has been argued that knowledge creation often takes place on the move [8]. This is especially true for urban planning, since planners frequently have to work in the field in order to assess the dimension of the problem on site [9,10]. Mobile computing and networking technologies can make a significant contribution in this type of scenarios providing tools allowing them to work outside the office.

Therefore, the main challenge of our research work is to integrate practical and theoretical aspects of visual geo-referenced data and information with a knowledge creation model, in order to use them as a basis for the design and construction of a software tool for mobile devices (Tablet-PC) with the goal of supporting urban planning activities in mobile scenarios combining face-to-face with computer mediated collaboration. Urban planning [2,11] and knowledge creation [5] necessarily involve various types of information and data related to the physical context or situation, such as the available physical infrastructure, the environment and landscape, land use, *etc*.

In order to develop a useful tool which can successfully support the work in the described scenario it is essential to identify and specify the necessary functionalities which enable the integration of geo-referenced data and information with knowledge creation processes. These functionalities need to be analyzed according to the following guidelines: (a) it is necessary to consider a knowledge creation model that allows its potential users to informally develop and maintain the necessary mechanisms to manage sense-making, knowledge creation and decision-making processes [12]; (b) in complex contexts such as urban planning, decisions are highly dependent on collaboration, which requires coordination, awareness and collaborative visualization support [13,14]; (c) knowledge creation in face-to-face mobile scenarios requiring geo-referenced-related data defines new types of brainstorming, talks, discussions, and negotiations requirements which should be seamlessly integrated in the tool [15]; and finally (d) according to the Socialization, Externalization, Combination, and Internalization (SECI) model, knowledge creation starts with the process of converting tacit knowledge into explicit knowledge through shared experiences during face-to-face social interactions [5].

We may therefore express with more accuracy that our main challenge consists in modeling a software application capable of geo-referencing data and information within the context of a broader model of the knowledge creation process, understood as a collaborative face-to-face endeavor. The users will be able to discuss about data and information automatically geo-located by the application, in the field. For the evaluation of the software tool we consider five requirements (labeled from R1 to R5) that should be fulfilled in order to effectively support collaborative knowledge construction. These requirements have been established by various theories and empirical research works, as well as the SECI model (see Section 3) about the conversion of tacit knowledge into explicit knowledge: **R1**, *support the spiral process of conversion of tacit into explicit knowledge* based on the SECI model [5]; **R2**, *support divergent and convergent thinking* [16]; **R3**, *support individual, dyadic, and group brainstorming and brainsketching* [17]; **R4**, *allows flexibility in the granularity of planning* [16,18]; and **R5**, *use of visual mechanisms, such as sketching and brainsketching* [9,19] to represent and convey knowledge.

The developed application will be tested against these requirements taking into account its three functional modes (labeled from FM1 to FM3): **FM1**, *brainwriting & brainsketching of ideas*; **FM2**, *relevant information selection*; and **FM3**, *visual representation of created knowledge*.

The paper is organized as follows: Section 2 presents a review of several concepts and definitions related to knowledge creation, sense-making, decision-making models, in order to highlight the main model constructs that support the design of a collaborative knowledge creation software application supported by visually-geo-referenced workspaces. Section 3 introduces a model for developing an effective knowledge creation-supporting tool for collaborative mobile scenarios and for tasks requiring geo-referenced information. Section 4 describes our proposal of a conceptual model for knowledge creation support. Section 5 describes the software application developed to explore the integration between knowledge creation and visually-geo-referenced data and information models. Section 6 presents an evaluation of the application, and Section 7 concludes the paper with a synthesis and discussion of the results obtained.

## 2. Knowledge Creation in Collaborative and Mobile Work Scenarios

### 2.1. The Strategic Role of Data and Information in Collaborative Work Scenarios

Studies emphasize three distinct areas in which the creation and use of data and information plays a strategic role when a group is collaboratively working to achieve any common goal [12]. First, any group of participants uses information to “make sense”. We understand sense-making as the principal information process for interpreting data, cues and messages about the environment. The outcome of sense-making is an ongoing series of enacted interpretations about the group members and their environment that constructs a shared context for action, or a reference frame for knowledge creation. Thereafter, participants generate new knowledge (knowledge creation). This knowledge is distributed among the group members and exists in different forms and venues. For example, individuals develop an informal kind of knowledge that is derived from practice and experience. In some cases this knowledge is tacit, non-structured information and must be extracted using knowledge acquisition and creation processes in order to be useful. Here, the main information process is the knowledge conversion. The creation and use of organizational knowledge, which simultaneously engages both tacit and explicit knowledge [20], emphasizes the conversion between the tacit and explicit through four processes: socialization, externalization, combination and internalization.

**Figure 1.**
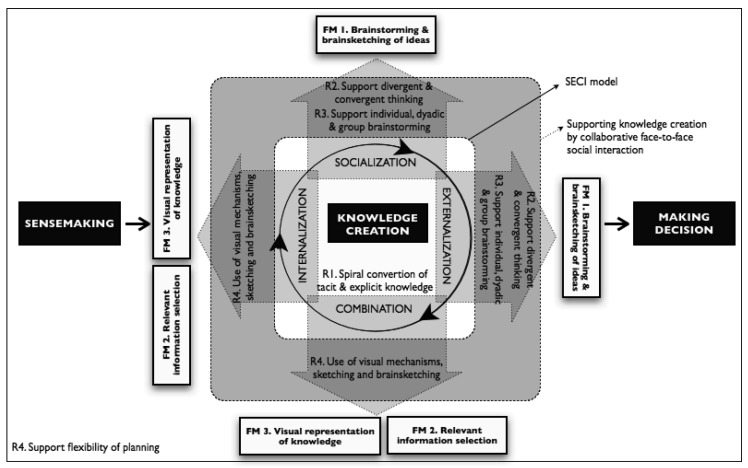
A conceptual model contextualizing the knowledge creation process support, based on the strategic role of information (sensemaking, knowledge creation and decision making). Each component of the SECI model is supported by requirements that support creativity, which are finally associated with the correspondent functional modes to be implemented on a software application supporting knowledge creation.

Finally, participants search for and evaluate information or knowledge created in order to make decisions. Here, the key activity is processing of information about available alternatives in order to select one that can achieve the desired objectives. In Figure 1 we can see the three concepts (sense-making, knowledge creation and decision-making) and their relationships with the knowledge creation process of the SECI model [20].

### 2.2. Situated Knowledge Creation in Face-to-Face Mobile Scenarios

In accordance to Grimm, Tazari *et al.* [21] and Thiele, Schader *et al.* [22], the ability to retrieve and store knowledge in mobile face-to-face working scenarios is crucial. Becerra-Fernandez, Cousins *et al.* [23] also indicate that knowledge is increasingly being created and applied on the move by knowledge workers who work jointly in face-to-face situations.

Some researchers have recently stated that the potential of knowledge creation is usually limited to stationary workplaces because most knowledge creation support systems are designed for use with Desktop PCs connected to a central server [4,22,23]. This excludes a multiplicity of mobile workers, many of them in charge of knowledge intensive activities. An organization's capabilities to support knowledge management may be extended through the introduction of mobile technology.

Therefore, we argue that mobile knowledge creation supporting situated mobile working scenarios has not attracted as much attention as it should, considering its potential. Researchers like Balfanz, Grimm *et al.* [24], Becerra-Fernandez, Cousins *et al.* [23], and Merckel and Nishida [4]) argue that mobile knowledge creation systems should be physical context-aware of the users' working situation and location. That is, a mobile face-to-face support system can be used as the basis to manage situated knowledge, *i.e.*, knowledge specific to a particular location. The key idea is to share explicit knowledge through interaction with the real world in order to allow users to develop tacit knowledge as well as acquire explicit knowledge.

The mobile added values concept refers to characteristics of mobile technology and its utilization in the sense of what can “mobile” do, what “stationary” cannot? (see [25] for examples). Derballa and Pousttchi [26] defined the following mobile added values characteristics: ubiquity is the possibility to send and receive data anytime and anywhere and thus eliminate any time and space restrictions. Context-Sensitivity refers to the delivery of customized data, location information or a service fitting the particular needs of the user in his/her physical location and current situation.

Physical context-sensitivity facilitates the knowledge creation process requiring explicit knowledge to be re-interpreted, re-created and appropriated on the field [14]. In many cases [13], knowledge needs a physical context to be created. Knowledge is context-specific [20], as it depends on a particular time and space [27]. Knowledge is created in a situated action [3,28], therefore, the knowledge-creating process is necessarily context-specific in terms of time, space, location and relationship with others. Knowledge cannot be created in a vacuum and needs a place where information becomes knowledge through meaningful interpretation. Nonaka and von Krogh [20] define a shared context in motion, in which knowledge is shared, created, and utilized and they call it *ba* (perhaps because ba means place in Japanese). *Ba* provides the energy, quality, and places to perform the individual knowledge face-to-face conversions and to move along the knowledge spiral. In other words, ba is a phenomenological time and space where knowledge emerges as “a stream of meaning”. New knowledge is created out of existing knowledge through the change of meanings and contexts.

### 2.3. Groupware in Geo-Collaborative Systems

This section discusses relevant concepts of groupware applications, and environments with collaboration in the geo-referencing of locations. We begin with the distinction between same-place and different-places, which has a focus on accessibility rather than geographical nature, determining the overall architecture and functionality of the system. Some subsequent developments of time (synchronous, asynchronous)/place map continue to emphasize the accessibility constraints. For instance, the expansion of the place dimension in three categories—co-located, virtual co-located and remote—, addresses the infrastructure capabilities to access each other in a team.

The conceptual change from place to space introduces a more broad concern with geographical relationships such as location, distance and orientation [29]. Places exist in spaces. Dix *et al.* [30] propose a taxonomy considering physical and virtual places, and Cartesian and topological locations. Analyzing the relationships between context, places and spaces, we find the distinction between private and public spaces, the former pertaining to things and actions belonging to one single individual and the later shared among members of a group [5].

The notion of virtual space is fundamental in Collaborative Virtual Environments [31]. Virtual spaces are interactive, shared, populated and may be navigated. According to MacEachren and Brewer, [2], interaction involves the aggregation of participants, topology of connections and dissemination of information. The navigation is not necessarily spatial but may also be logical. Virtual spaces may assume complex structures, such as clusters, stacks, lists, tables, rooms, *etc.* Then users should be able to navigate these structures.

Dix *et al.* [30] propose various levels of mobility: fixed, mobile, autonomous, free, embedded and pervasive. Herman [16] studied the relationships between mobility, location awareness and location services to derive important requirements such as flexibility, visibility and context-sensitivity. Collaborative visualization, as an enabler of interaction and collaboration, is naturally another major challenge to consider in virtual spaces [6]. Collaborative visualization involves at least data exchange, shared control and dynamic interaction [2].

We should also analyze the notion of workspace. According to [31], a place has inherent a set of activities that occur there, while a workspace is just a container of places with ongoing activities. We may distinguish two categories of workspaces: structured and geo-referenced workspaces. The structured workspace organizes (logically or physically) several activities in coherent sets, which are nevertheless independent from the place itself. A group editor is a good example of this type of workspace, since the workspace serves to organize different activities, like writing and revising, while maintaining a coherent view of the whole [32]. A geo-referenced workspace organizes activities dependent on the geographical place (physical context or location) where they are carried out.

Recent exploratory works present geo-collaborative applications or prototypes having a geo-referenced workspace component to solve crisis management, decision making, and knowledge acquisition problems.

#### Crisis Management

The system described in [27] supports synchronous and asynchronous interaction among users working in different places, providing geo-referenced localization services. The system presented in [33] uses mobile devices to provide synchronous interaction among users located in different places; The application presented in [11] uses mobile devices to support synchronous and asynchronous interaction among users in various places.

#### Decision-Making

In [34] the authors present a system supporting synchronous navigation of distributed users in a virtual co-located space; The work presented in [35] supports the synchronous visualization of social interactions located in the same workspace through co-located mediation.

#### Knowledge acquisition

Convertino, Ganoe *et al.* [6] present an application using non-mobile technology supporting synchronous interaction among users located in various places while working in a virtually co-located workspace.

Although the physical context-situation (location) is an important component of knowledge, there is no evidence in the literature about systems taking advantage of mobile and GPS technology to support a group of users in knowledge creation activities using this information. In this paper we present a geo-collaborative mobile, co-located and visual system supporting knowledge creation for designing and planning, using mobile devices (Tablet-PCs) equipped with GPS allowing a group of users to synchronously work over a common virtual workspace. It makes use of geo-referenced information over maps in a face-to-face scenario helping users to share their tacit and explicit knowledge.

Geo-collaboration is an emerging activity where users explore geospatial information through geo-referenced information [13,33,36], in order to solve problems requiring a workspace and location components to be represented. Some examples are land suitability evaluation, plan/scenario evaluation, site search/selection, resources allocation, location-allocation or impact assessment [15]. This form of collaboration can occur in both co-located and virtually distributed settings. These systems support virtually or real co-located group knowledge creation for decision-making and planning using geographic visualizations to explore the initial available information and view intermediate results. Geo-collaborative systems have been used mainly to communicate planning scenarios and outcomes [13]. Their system design includes a digital workspace for map-based analysis and visualization, multi-modal interfaces for allowing interactions between participant with various roles, and databases to provide baseline data and store new information.

## 3. Supporting Knowledge Creation by Collaborative Face-to-Face Social Interactions

According to Nonaka and Toyama [5], knowledge creation cannot be independent from the individual's own context. Social interaction, spatial, cultural, and historical contexts are important for individuals, because such contexts give the basis to interpret information to create meanings. Furthermore, their knowledge creation theory is conceptualized as a dialectic process where new boundaries are created through the social interaction among people. This dialectic process, driven between tacit and explicit knowledge, is explained by the SECI model as taking place in four processes, socialization, externalization, combination, and internalization [20], which proceed one after another in a spiral way. Therefore, the first requirement we will consider for designing the knowledge creation tool is based on the SECI model of Nonaka and Toyama [5]. The tool should support the *spiral conversion of tacit and explicit knowledge*, hereinafter also referred to as **R1**, in which knowledge creation starts with the process of converting tacit knowledge through shared experiences in face-to-face social interaction into explicit knowledge, which is amplified in a spiral way through the four processes of knowledge conversion: socialization, externalization, combination, and internalization.

Additionally, it is widely agreed upon that the creativity of ideas increases if they are developed collaboratively and in a face-to-face situation [16]. Many face-to-face collaborative tools supporting the collaborative creative process have been developed [9,16,18]. They take advantage from bringing together people and that the spatial, temporal, cultural, and technical distances between them, as well as conceptual collisions, enrich collaboration. Therefore, we will consider four more requirements for supporting creativity which derivate from these diverse theoretical and empirical investigations. The tool should: *support divergent and convergent thinking* or **R2**, this means it should support the process of generation of different alternative ideas, as well as the process of choosing an alternative that fits the problem, [16]; *should support individual, dyadic, and group brainstorming* or **R3** by providing the proper mechanisms such as private and public workspaces [17]; *support flexibility in the granularity of planning* or **R4** [16,18]; and make *usage of visual mechanisms, such as sketching and brainsketching* or **R5** [9,19].

In the following five subsections we discuss each one of these five requirements in more detail and focused on collaborative geo-referenced knowledge creation in order to incorporate them in a conceptual model of knowledge creation support over which the developed application is based.

### 3.1. Spiral Conversion Process of Tacit and Explicit Knowledge

Nonaka's SECI model for knowledge creation [20], includes four knowledge transformation processes. The first one is called *socialization* (tacit-tacit—*sharing and creating tacit knowledge through direct experience*) and refers to the process of knowledge assimilation and its conversion to a new tacit knowledge among individuals who experience face-to-face collaboration. Here, knowledge is transferred by demonstration, observation, apprenticeship, behavior modeling, actual practice or, doing. The second is called *externalization* (tacit-explicit—*articulating tacit knowledge through dialogue and reflection*) and refers to the conversion of tacit knowledge into explicit knowledge, which occurs when tacit knowledge is described or abstracted as concepts, formulas, rules and theorems, etc, directly with the spoken and written languages, observation, walk tracking, “osmosis”, *etc.* This process occurs in groups and communities. Knowledge is transferred through (partially) explicit information by the use of metaphors, analogies, prototypes or sketches. The third one is called *combination* (explicit-explicit—*systemizing and applying explicit knowledge and information*) and refers to the production of new explicit knowledge through analyzing, classifying and sharing of explicit knowledge. In this case, knowledge is transferred formally and informally, by verbal or written means. The fourth one is called *internalization* (explicit-tacit—learning and acquiring new tacit knowledge in practice) and refers to individuals or organizations applying theory to practice, turning explicit knowledge into one's own tacit knowledge through practice.

According to this, the SECI model can be applied to explain scenarios where people are working face-to-face generating knowledge by collaboratively geo-referencing data and information over maps. By this mean, participants can convey their tacit knowledge by making them explicit through sketching over map, along with annotations, for example in order to share their ideas about which new streets should be built and how will they run, or which will be the shape of a new green area, which area will it cover. Doing this activity on the site where the street or park should be built allows them to include geo-referenced data automatically as well as important context information, which maps may not convey.

### 3.2. Support Divergent and Convergent Thinking

Creativity, in any domain, involves both divergent and convergent thinking [16]. Divergent thinking is the ability to generate a set of possible responses, ideas, options, or alternatives to an open question, task, or challenge. Because the process of creativity involves a continuous interplay between divergent and convergent thinking, we do not treat them separately. Instead, based on the literature, we illustrate different ways in which both divergent and convergent thinking can be facilitated, and, eventually be supported through technology.

Urban planning requires the collaborative work of various actors, like architects, urban designers, city planners, and public administrators; they require at the end to make decisions based on consensus. Their proposals are the result of tasks requiring discussion and agreement, based on diverse criteria in order to analyze opportunities and threats, advantages and drawbacks, benefits and costs of the proposed ideas.

### 3.3. Integrated Support for Individual, Dyadic, and Group Brainstorming

During the creative work stage, group members alternate between individual, pair-wise, and work group. Therefore, supporting these different brainstorming modalities and the alterations between them seems a plausible and feasible idea. Maintaining history of brainstorming sessions, which would be bookmarked when modality switching occurs, would allow users to refer back to previous versions, assess changes temporally, and keep track of who did what. Such session histories would facilitate the meta-cognitive process of reflection and self-awareness, and establishment of a reward structure for making work visible. Brainstorming techniques—such as drawing concept maps, affinity diagrams, or storyboarding—are often codified as graphical visualizations of knowledge. One way to integrate support for individual, dyadic, and group brainstorming is to use role-specific multiple view visualizations [17]. Multiple view visualizations could then possibly represent different perspectives on how a problem should be broken down. For example, using the notion of public and private spaces, an individual could first develop ideas privately, and later propagate these ideas to the group through the shared view during a brainstorming session.

The various actors involved in urban planning usually require to work according to different modalities: in some cases, due to the expert knowledge of one each one they have to work individually before sharing their ideas with the rest. If more than a single expert on a particular subject are joining the group they may start working together as a subgroup before joining the work with the rest of the group. Finally, at a certain stage a collaborative work session involving all participants will be most probably required.

### 3.4. Support Flexibility in the Granularity of Planning

Although more detailed plans can lead to creativity, imposing such constraints in collaborative systems can be problematic. To be too rigid can potentially stymie creativity, and users often find ways to work around them. In [18] the authors argue that a flexible, more opportunistic and less imposing, planning tool allowing various levels of detail would facilitate creativity. Planning can be conceptualized as strategic and operational. Separating and supporting different levels of planning may bring flexibility in planning tools.

When performing an urban planning activity solutions might be attained in various ways. Some would require long working session's performing convergent and divergent tasks, other may require just analyzing facts and compiling information. Therefore, in urban planning there is no pre-defined working methodology. Therefore, a system supporting urban planning work should be very flexible regarding this issue.

### 3.5. Usage of Visual Mechanisms, Sketching and Brainsketching

According to Yongjin, Xinyan *et al.* [9], systems using visualization mechanisms to manage information facilitate the conversion of tacit into explicit knowledge. For them, visualization enables knowledge “mapping” facilitating its creation and sharing. In knowledge creation visualization is used to support the creation of tacit knowledge individually or collaboratively by means of sketches, concept maps, graphical representations, *etc.* It facilitates the clarification and enrichment of tacit knowledge for an individual himself or when trying to share that knowledge with others, supporting the development of different points of view. Van der Lugt [19] highlights the following advantages of sketching in idea face-to-face generation meetings: (a) thinking—sketching stimulates a re-interpretative cycle in the individual participant's idea generation process; (b) talking—sketching stimulates the participants to re-interpret each other's ideas; and (c) storing—sketching stimulates the use of earlier ideas in the idea generation process by enhancing their accessibility. The visualization technique called “brainsketching” [19] was used to describe idea generation techniques that use sketching. Van der Lugt suggests that this process should be supported by an electronic medium which visualizes and combines all participants' contributions and allows them to develop all kinds of ideas, opinions, illustrating material or contextual background information into a large picture.

Visualization, sketching and brainsketching are useful concepts to be included into in geo-collaborative systems. They often include a digital workspace for map-based analysis and visualization; multi-modal interfaces enabling interactions between users.

Regarding sketching and brainsketching as visual mechanisms, it is possible that at an early stage of the planning work ideas proposed by a person might not be very precise and clear. By means of sketches a simple and general representation of these preliminary ideas can be expressed and communicated in a visual way (brainsketching). Additionally, by working face-to-face verbal explanations could be provided in order to complete the ideas represented by a simple sketch without needing complex and detailed representations, [10]. It is also frequent that people working with paper maps make freehand annotations over it in order contextualize the discussion to a certain region on the map, specify information associated to a concrete location on the map on the fly, or to mark physical zones with new information e.g. sketch a new road or re-design green areas. [9].

## 4. Conceptual Model for Knowledge Creation Support

Considering the five requirements for supporting knowledge creation presented above, in this section we propose a conceptual model which identifies and supports the essential components and basic functionalities a software application supporting knowledge creation in mobile geo-collaboration scenarios should have.

In the first place we consider the **R1** requirement *spiral conversion of tacit and explicit knowledge*, as the fundamental part of our model. As we mentioned before, the model incorporates the four processes of the SECI model explaining how knowledge is created and converted in a spiral way by individuals, a group of individuals and by the interactions among various groups inside an organization (see Figure 1, the three arrows at the center of the figure represent the spiral cycle between the processes of the SECI model). In the context of our proposal, in the spiral cycle of knowledge the conversion and interaction between tacit and explicit knowledge takes place in a synchronous collaborative and co-located context while working face-to-face. Knowledge emerges from reflection, social face-to-face interaction and the dialectic's dynamic, while individuals work on the move. The created knowledge is associated to the concrete physical location where it emerges. The model is aimed at supporting scenarios where geo-referenced data is an important component of this knowledge.

Along with the knowledge creation process, the model also considers the processes that take place before (*sense-making*) and after (*decision-making*) [12] in a broad sense. The model considers providing the user with the relevant information in order to understand and give a meaning to the activity they are performing. It also considers sharing this information in order to build a common understanding of the situation. In a similar way, the model considers providing users with the necessary tools to manage the various alternatives that may be generated during the knowledge creation process.

In the second place, we consider the other four requirements: **R2**—*support divergent and convergent thinking*; **R3**, *support individual, dyadic, and group brainstorming*; **R4**—*support flexibility in the granularity of planning*; and **R5**—*Usage of visual mechanisms, such as sketching and brainsketching* which have close relationships with the four processes of the SECI model. The model associates the requirements with the processes of the SECI model as can be seen in Figure 1. This association introduces two important advantages: it allows the definition of a simple conceptual model and it facilitates the identification of the functionalities the application should implement. **R2** is associated with socialization and externalization because it is precisely in those processes where the dialectic dynamic, reflection and social interaction take place, which are necessary to create and share tacit knowledge (socialization), and articulate them through dialog and reflection (*externalization*). **R3** is also associated to *socialization* and *externalization*, since these processes can be implemented through individual, dyadic or group brainstorming. **R4** is associated with all processes of the SECI model since knowledge creation is a spiral process which does not requires a specific sequence nor a specific starting or ending point. **R5** is associated with the *internalization* and *combination* processes since the use of visualization, sketching, and brainsketching are oriented to represent, reinterpret, and internalize other's ideas (*internalization*) and to visualize, combine, and share them in order to facilitate their understanding (*combination*).

In the third and final place we include in the model the necessary functional modes a knowledge creation supporting tool should have. We propose three functional modes for our scenario: **FM1**—*brainstorming & brainsketching of ideas*; **FM2**—*relevant information selection*; and **FM3**—*visual representation of knowledge created*. Each one of these functional modes corresponds to specific requirements of knowledge creation. **FM1** is associated with the *socialization* and *externalization* processes of **R1**, as well as with the **R2** and **R3** requirements, (this is represented by the area in the shape of an up-going vertical arrow and a right-going horizontal arrow in Figure 1). **FM2** and **FM3** are associated with the *internalization* and *combination* processes of **R1** and **R5**, (this is represented by the area in the shape of a left-going horizontal arrow and a down-going vertical arrow in the Figure 1). The specification of the software implementation of each functional mode depends on the requirements of knowledge creation to which they are associated. This is described in the next section.

## 5. Description of the Software Application

The software application is a collaborative situated knowledge creation tool supporting urban planning and it is based on the knowledge creation model presented in section 4. According to this, the system has three functional modes: **FM1** mode integrates for divergent and convergent thinking support as well as individual, dyadic and group brainwriting & brainsketching; **FM2** mode implements visual mechanisms to rank and/or select ideas; and **FM3** mode, which is associated to the *internalization* and *combination* processes introduces the advantages of sketching and the use of visual mechanisms.

The three functional modes of the software application are aimed at providing an environment facilitating tacit knowledge sharing, transferring and creation, offering the possibility to do this without the need to convert tacit knowledge into explicit knowledge before sharing it. Hence, face-to-face communication should be used to share and personalize tacit and explicit knowledge, rather instead of extracting, coding and storing it. The sharing of tacit and explicit knowledge is viewed as a social process between individuals requiring face-to-face interaction while working in mobile scenarios to achieve urban planning tasks.

When the application is started an *ad-hoc* network is automatically established among each of the participant's Tablet-PCs. The first view of the interface is the main window which displays a workspace containing a map of the current physical location the users are currently located. Beside this workspace, three other windows are displayed, each one belonging to each of the three modes the system supports, the upper right window corresponds to the **FM1** mode, the middle right window to the **FM2** mode, and the bottom right window to the **FM3** mode. Since the map of the main window corresponds to the physical area where users are, if they move to another physical location the map moves with them. This is achieved thanks to the GPS of each Tablet-PC. If users require geo-referencing other locations on the map, they can activate a mode that allows them to visit other parts of the map different from their current location.

The application allows geo-referencing data and information through the following mechanisms: (1) directly drawing sketches over the map; (2) creation of recursive concepts maps; and (3) creation of localization marks over the map. All these operations are possible while working in any of the three functional modes thus introducing more flexibility to the activity. Thanks to this, the application can be used in various ways allowing participants to (a) collaboratively geo-reference only the necessary information without using any of the three modes, (b) use the **FM1** mode (individually, dyadic, or in a group), while associating the geo-referenced sketches and annotations over the map; (c) have a space to express divergences and convergences of ideas using the **FM2** mode while referencing the information over the map at the same time; or (d) create a visual presentation of relevant ideas using gestures and sketches using the **FM3** mode. In the same way, all three modes can be used independently or combined, according to the users' needs, which fulfils the requirement **R4**—*support flexibility in the granularity of planning*. In each mode the interface shows the geo-referenced information on the map.

**Figure 2.**
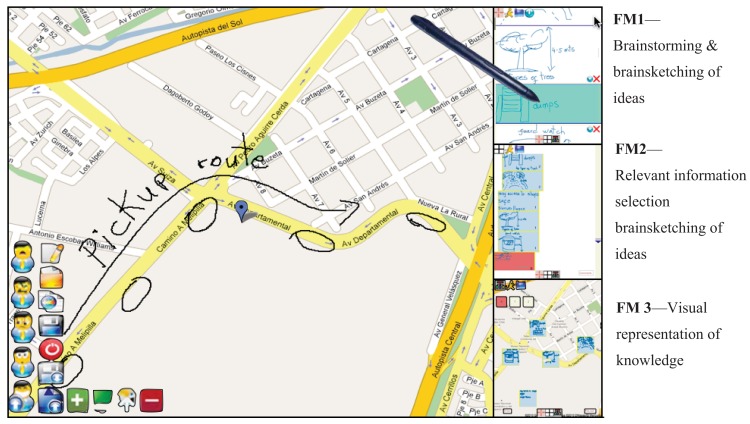
Main human computer interface of the application. The **FM1**, **FM2**, and **FM3** modes are at the right column. The figure shows the case of how an idea corresponding to the design of a dump edited in the mode brainstroming & brainsketching and selected by the Tablet-PC pen has been associated to sketches specified on the map, indicating where the dumps should be located. In the mode of relevant information a list of many ranked ideas can be seen. In the mode of Visual representation of knowledge creation it can be seen the ideas selected and associated to the map where the place they should be applied is geo-referenced.

The geo-referenced information can be associated to a certain location on the map by selecting the geo-referenced information and then the corresponding idea in any of the three modes. It is also possible to scroll and zoom over the map, store and load the information. These functions are activated trough the menu located on the left-bottom of the screen (see Figure 2). By default all users work individually, though the peer-to-peer connection among them is always established and they can see other users in a close environment through icons representing them. The icons located in the left-down corner of the screen allow switching among individual, dyadic and group work, as well as selecting the users to work with. If a user “A” requires to collaboratively work with another user “B”, user “A” should click on the icon of the interface of user “B”; user “B” will receive this request in his/her interface as a message on the icon of user “A”. In order to accept the invitation to work collaboratively, user “B” must click on this icon.

**Figure 3.**
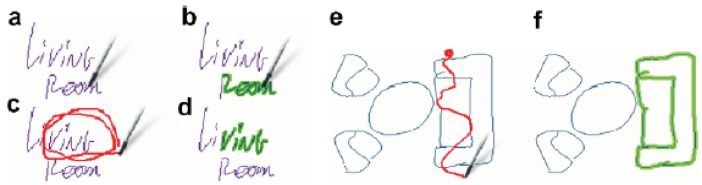
(**a**) After creating a sketch, holding the stylus on a stroke (“room” work on the example) will (**b**) select connected graphics (partially darkened “r”, and “oom” on upper right). (**c**) Double-surrounding (“ving” on lower left) will (**d**) select enclosed traces (darkened “ving” on lower right). (**e**) Drawing a dense dot and then moving the stylus over other traces will (**f**) select all touched elements.

Editing actions are executed using gestures over the working area (map): Selecting sketches is done by any of the following three gestures: The first one consists on clicking with the pen on a given trace or line (see Figure 3(a)), which will select all other traces touching it (Figure 3(b)). The second is to double-surround them with a continuous closed shape (Figure 3(c,d)). This allows the selection of a group of not necessarily connected strokes. The last method is used as an alternative to the double-surrounding and consists of drawing a dense dot and then, without releasing the stylus, drawing a line which touches the different elements that the user wants to select (Figure 3(e,f)). This last method also copies the selected strokes into a clipboard (see Pasting below). The different methods help the user to select of a group of strokes more easily under different scenarios: for instance, double-surrounding is easy and fast for complex drawings (like writing), while dot-selection is faster for selecting strokes in large drawings.

Deselecting is done by clicking on any empty space. Using any of these methods more than once in succession will add or remove items from the selection so that the user can make complex selections using simple gestures.

Pasting is done by drawing a dense point and releasing the stylus. This duplicates elements previously copied into the clipboard, and places them where the point has been drawn. When one or more items have been selected, two small handles appear at the right side of the selected strokes.

**Figure 4.**
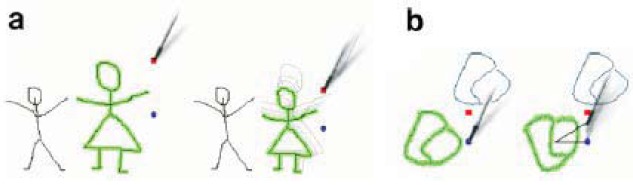
(**a**) Resizing selected strokes by dragging the red square handle. (**b**) Rotating selected strokes by dragging the blue round handle. These handles are always shown when a sketch has been selected, but they have been removed from other figures in this paper to simplify understanding of explained features. (For interpretation of the references to color in this figure legend, the reader is referred to the web version of this article.)

There is a set of simple actions for editing selected shapes: Moving is accomplished by dragging any selected stroke or sketch. Dragging will move the selection as a whole. Resizing is done by dragging the red square handle, located at the upper right corner of the selected group of elements (see Figure 4(a)).

**Figure 5.**
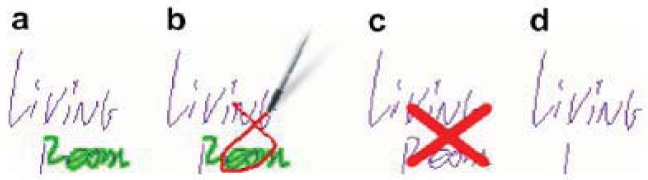
(**a**) When a strokes is selected; (**b**) a connected cross gesture triggers the Remove command; (**c**) feedback appears when command is recognized and (**d**) only selected strokes are removed.

Rotating the selected elements can be achieved by dragging the blue round handle, located at selection's right size (see Figure 4(b)). Removing is performed by drawing a “connected cross” (see Figure 5). If nothing is selected, this gesture removes every touched element. If one or more traces are currently selected, only those elements will be removed. Now we will describe with more detail each of the three implemented working modes.

### 5.1. FM1—“Brainstorming/Brainsketching of Ideas” Functional Mode—Socialization and Externalization SECI Model

**Figure 6.**
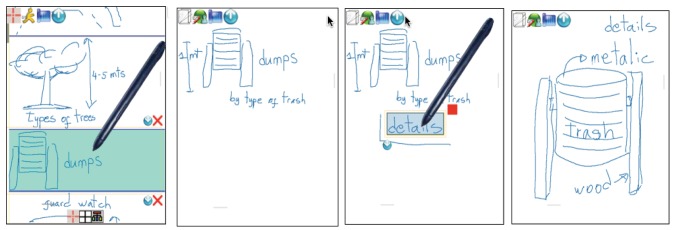
Editing ideas through sketching and freehand writing in the brainstorming/brainsketching mode. From left to right: in the first screenshot an idea consisting of the design of a dump is generated with a brief explanatory text, this idea is separated from the rest by making a gesture corresponding to a horizontal line. To edit this idea, it is necessary to select it with a click of the pen and enter clicking the icon with the arrow down. In the second screenshot the idea previously selected is being re-edited by adding more details. The third shows how to generate a node through a gesture in the shape of a rectangle. This node is now a new sub-idea within an idea, which can also be edited by selecting and clicking on the icon with the arrow down, as shown in the last screenshot of the sequence, where details of the trashcan are.

This mode supports the knowledge externalization allowing users to explain their tacit or explicit knowledge by means of freehand writing or sketching. This mode allows users to freely prepare their ideas before sharing them, reducing the free-riding, production blocking, and evaluation apprehension problems [37]. Users generate their ideas in parallel despite they are in a face-to-face situation (see Figure 6). If a previous idea has to be edited, the user selects it by a single click and “enters” the edition mode clicking the “arrow down” icon (left of Figure 6). This supports the knowledge socialization process of the SECI model. Since ideas are shown one below the other a scrolling function is necessary to go through them, which is done by a gesture of sliding the stick up and down parallel to the right vertical border of the screen.

Every idea specified in this mode can be associated to the specific localization where users are working, geo-referencing data and information by sketching and freehand writing on the map as shown in Figure 2. As new ideas are being edited, there is a new map in the main window associated to the physical location where users are located. In order to go back to data and geo-referenced information on the map, the user simply selects the idea previously defined.

### 5.2. FM2—“Relevant Information Selection” Functional Mode—Internalization and Combination SECI Model

**Figure 7.**
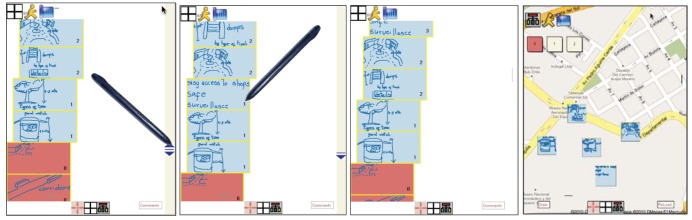
On the left, scrolling the ideas by moving the pen on the right border of the screen. The second and third screenshots show how a sketch can be converted in a node. At the right all ideas of level 1 were used on the knowledge creation representation.

After each user has externalized her ideas individually or collaboratively, it is necessary to analyze them involving all group members in order to select the most relevant ones and/or discard the irrelevant ones. To support this process, the system generates a list of all created ideas, which will be visually shown as rectangular boxes of similar proportions with colors associated to the participant who has created them. In this stage, the list of ideas is visible to all participants, as shown in the Figure 7. In order to rank them, participants have to vote for them positively or negatively. They can issue a positive vote for a certain idea by making a tick gesture on the left area of the rectangle representing it (see the middle of Figure 7). A negative vote is issued by making a tick on the right area of the rectangle. Numbers from 1 to 5 represent the ranking of each idea according to the votes received, being 5 the most relevant. Because there might be many ideas, a scroll mechanism is also available in this mode (left of Figure 7).

At the beginning, before receiving any vote, the ranking number for an idea is 1. This ranking number appears at the bottom-right corner of the rectangle. As ideas get ranked, they will be automatically rearranged and grouped according to the ranking number. In this way, relevant ideas are easily distinguished from the irrelevant ones, supporting their selection.

An idea can be collaboratively edited while working in this mode by clicking in the middle area of the rectangle. Collaborative editing allows the socialization of the tacit and explicit knowledge, allowing participants to combine their knowledge and perspectives about the ideas. It is possible to add more information to an idea by means of including concept maps. Concept map's nodes can be created inside an idea by making an “L” gesture enclosing a single or a group of strokes previously drawn (from left to right, see second and third screenshots of Figure 6). Users can get inside of these nodes to include more information as well as to recursively define new nodes, thus creating a hierarchy of nodes. These conceptual maps allow users to organize and add a semantic meaning to the information.

### 5.3. FM3—“Visual Presentation of the Knowledge Created” Functional Mode—Internalization and Combination SECI Model

This mode allows users to summarize the knowledge creation process using a final visual representation of the ideas. This process is done collaboratively with the agreement of all participants. In this mode the ideas selected in the previous mode are arranged over the map they are geo-referenced.

At the beginning of this mode, a map of the localization where the users worked appears with a list of small squares at the top representing the generated ideas ordered according to their ranking. In this stage, participants have to make a visual arrangement of the ideas. This is an important stage during the knowledge creation because it is expected the tacit and explicit knowledge to be expressed here with sketches and other visual representations. It is expected that participants first draw a sketch where ideas will be placed in a particular order according to the meaning of the sketch. Ideas can be dragged from the list and dropped in the desired place. The placement of the ideas inside the sketch should represent a meaning collaboratively defined by all participants. The rectangle representing an idea can be reshaped as desired (last screenshot of Figure 7). After placing the ideas on the schema, participants may finalize their proposal by skating which one would be fundamental to the project or they can go back to a previous mode in order to edit the existing ideas or include new ones. Not used ideas might be deleted.

## 6. Evaluation of the Software Application

### 6.1. Issues in Collaborative Systems Evaluation

The success of a collaborative system depends on multiple factors, including the group characteristics and its dynamic, the individual, social and organizational context in which it is inserted, and the positive and negative effects of technology on the group's tasks and processes. Therefore, collaborative systems evaluation is always necessary to determine the impact a software solution will have on the individuals, groups and the organization. According to [38] several evaluation methods have been proposed, which comprise a variety of approaches with various goals. Ideally, a single evaluation method should cover the individual, group and organizational domains, assessing whether or not the system is successful at the combination of those realms. Unfortunately, no such single method is currently available, and may never be. The fundamental cause for it is related with the granularity and time scale of the information obtained at these three domains: the information pertaining to the individual is usually gathered at the cognitive level, focusing on events occurring on a time frame in the order of a few minutes or even seconds; group information is gathered at the interaction/communication level, addressing activities occurring in the range of several minutes and hours; and the information regarding organizational impact concerns much longer time frames, usually in the order of days, months and even years.

### 6.2. Evaluation Scenarios and Guidelines

Three evaluation scenarios (role-based, rule-based and knowledge-based) were proposed in [38] jointly with a set of guidelines to select the appropriate evaluation methods to evaluate collaborative systems according to the following parameters: realism, generalization, precision, system detail, system scope and invested time. In the role-based scenario, the evaluation data is gathered at the individuals' cognitive level, focusing on events occurring during a time frame in the order of minutes or even seconds. The most adequate evaluation methods to employ in this scenario adopt laboratory settings and considerable instrumentation: human performance models, and performance analysis. In the rule-based scenario, the evaluation data concerns several subjects who must coordinate themselves to accomplish a set of tasks. The relevant events now occur over several minutes and hours, instead of minutes or less. The evaluation methods employed in this scenario may still adopt laboratory settings although using less instrumentation: cooperation scenarios, groupware observational user testing, and groupware heuristic evaluation. Finally, in the knowledge-based scenario, the evaluation is mostly focused on the organizational impact and thus concerns much longer time frames, usually on the order of days, months and even years, since the technology assimilation and the perception of value to the organization may take a long time to emerge and stabilize. The evaluation scenario is also considerably different when compared to the other scenarios, involving for instance knowledge management, creativity and decision-making abilities: cooperation scenarios, scenario-based evaluation, perceived value and “quick and dirty” ethnography.

According to the guidelines proposed in [38] selecting an evaluation method depends on the development status of the collaborative software application being assessed. For an already implemented software application the recommendation is to use of a knowledge-based method, to understand if the system functionality matches the goals and purposes. The knowledge-based evaluation is naturally most adequate to products giving latitude of decision to the users and supporting interaction, collaboration and decision-making.

### 6.3. Knowledge-Based Evaluation of the Application

As explained before, the goal of the developed application is supporting knowledge creation during urban planning activities in mobile, collaborative scenarios. In order to evaluate if this goal has been achieved we consider the variables corresponding to the five requirements for knowledge creation analyzed in Section 3, associated to the functionalities implemented by the application described in Section 4. The evaluation was focused on assessing the value the software application brings to the evaluators. We adopted the cooperation scenario method (COS) [39], where evaluators conduct field studies, semi-structured interviews, and workplace visits. They thus identify scenarios, collaborative behavior, users involved in it, their roles and the relevant context. For each role involved in the collaborative activity, evaluators analyze the software design to see how the task changes and who benefits from the introduction of the new technology. Then, the software application is presented in a workshop with users to discover design flaws.

The evaluation procedure was set up as follows. The tool was evaluated in three pilot experiments scenarios performed in real mobile working scenarios involving four, four and six evaluators, respectively. Each pilot experiment scenario was performed separately and independently of each other. Also for each evaluation different evaluators were used. All of the evaluators were knowledgeable in individual urban planning design, with professional experience on urban planning, and “computer literates”. Each pilot experiment scenario started with a brief tutorial about the tool, which took approximately 20 minutes. Then, the reviewers used the software application until the task was achieved, which consisted in collaboratively performing a specific urban planning task which included the need of handling geo-reference data and information. During the experiments, whenever necessary, additional help about the tool was provided to the evaluators.

In the first experimental scenario two city planners with two and six years of work experience and two urban designers with three and 12 years of work experience participated, one female and three male. We proposed them the task of planning the construction of a new parking place for the most important soccer stadium in Santiago de Chile. They had four working sessions of 30 to 40 minutes each, performed over two weeks, during which they used the prototype in order to collaboratively work in a face-to-face modality to generate the ideas for the location and characteristics of the parking place. The second experimental scenario was performed with two architects with five and nine years of work experience and two public administrators both with 10 years of work experience, again one female and three male. We asked them to perform the task of designing evacuation ways for an area located in the south-west part of Santiago of Chile where many new buildings are going to be built replacing old one-storey constructions (see Figures 2, 6, 7). Similar to the first group, they had four working sessions of 30 to 40 minutes each, performed over two weeks. The last experiment scenario was performed with three city planners with three, four and six years of work experience and three architects with four, five and eight years of work experience, two female and four male. We asked them to perform the task of re-designing pedestrian and motorized accesses, designing new areas for children recreation, water fountains, *etc.*, for a big public recreational park which covers an area of approximately 20.000 sq. m. They had two working sessions of 80 and 60 minutes each one over one week.

After the last session of each experimental scenario, we asked the evaluators to complete a semi-structured interview about the application's most positive and negative aspects, as well as to answer closed questions concerning the software's utility and usability.

### 6.4. Evaluation Results

Regarding the utility of the application, each one of the application's functionalities (see Sections 5.1, 5.2 and 5.3) and their association to the corresponding requirements (see Sections 3.1 to 3.5) were explained to the evaluators with the purpose of enabling an objective evaluation of the benefits the application brings to knowledge creation and to eliminate the ambiguity during interviews and questionnaires. For each functionality and its associated requirements (**FM1** associated to **R1**, **FM1** associated to **R2**, **FM1** associated to **R3**, **FM1**, **FM2** and **FM3** associated to **R4**, **FM2** associated to **R5**, and **FM3** associated to **R5**), one or two semi-structured questions were designed and one or zero closed questions, in order to identify and evaluate the level of benefit the evaluators considered the functionality provides to the knowledge creation process in terms of a value on the Likert scale. Answers of semi structured interviews were transformed into a value of the Likert scale. Closed questions used the Likert scale directly. The results are shown in Table 1.

Regarding the usability of the application, three characteristics were evaluated: comprehension or understanding of the application, how easy was to learn how to use the application, and operability related with the effort controlling the urban design collaborative task. In a similar way, one or two semi-structured and closed questions were designed for each characteristic. The results are also shown on Table 1.

After each situated experimental scenario we conducted scenario-based workshops where all reviewers had to analyze the application in the context of the predefined scenario. From the workshop we finally obtained a set of comments and observations regarding the utility and usability of the software application explained in the next paragraphs.

Regarding utility issues, the obtained results indicate that according to the evaluators' view, the application was easy to use and useful to support knowledge creation for situated urban planning design tasks in mobile working scenarios. We obtained positive indications about the three functionalities **FM1**, **FM2**, and **FM3** for each pilot experimental scenario. The results on Table 1 associated to each one of the three experimental scenarios show more or less similar results which ratifies that diverse evaluators with varied tasks had a similar the perception about the ability of the application to support knowledge creation. In the second experimental scenario the evaluators' results were on average not as good as the results given by evaluators of the other two scenarios. One possible explanation for this result is that this group was the one with less exposure to the application, so they might have not enough time to learn how to fully make use of it. The users indicated that sketches (**FM1**) helped to exteriorize and share tacit knowledge, and the visualization (**FM3**) of the artifacts on the system interface associated to data, information and functionalities triggered by gestures was highly useful. Users considered that sketches on the map can be easily associated to the ideas generated individually as well as collaboratively. They considered useful that a map of the location where they were working was shown in every moment. The possibility of re-editing the ideas already generated (Figure 6—dump design), as well as generating more nodes (concept maps) that contain details of an idea proposed were highly praised (the last two screenshots in Figure 6). While, they stated that at the beginning they perceived some complexity in the associating generated ideas to locations, they indicated that this feature allowed them to organize all the ideas they had regarding to different aspects of the solution in a geo-referenced manner (design of exit areas for vehicles, pedestrian corridors, garbage bins, security cabins, surveillance cameras, *etc.*). All except three users stated they agreed that the application allowed them to associate, express and reveal their ideas in the context of the activity performed and the physical place where they were located. All agreed that sketching and freehand writing as well as geo-referencing data facilitated the explanation of their proposed solutions and the decision making process. The evaluators positively assessed the possibility of collaboratively selecting and classifying the proposals (**FM2**). This allowed them to have a fast access to the various proposed options, while at the same time keeping these options associated to the location where they were generated. The flexibility to choose any one of the three functional modes of the application allowed them to introduce various and varied alternatives, since sometimes it was not necessary to use all functional modes (for example, they cited the **FM2** and **FM3**) because the face-to-face coordination was enough to accomplish the task supported by those modes. This makes us think that the functional mode the evaluators most appreciated was **FM1**.

Concerning usability issues, the obtained results indicated that participants could understand the working logic behind the application and that they effectively learned to deal with its functionality, as well as with the knowledge creation processes. However, it was pointed out that the application was a bit difficult to use at the beginning. Some other minor functional and user interface details were also raised by the evaluators, e.g., the absence of graphical information and the difficulties obtaining a summary view of the alternatives, and in some cases the lack of information about the authorship of the sketches. More experimented user missed the menus, choice boxes and fast access keys. The evaluators considered a major challenge to keep the awareness information and collaboration constantly up-to-date. The learning curve of the application was satisfactory completed during the second working session for most of the evaluators. In relation to the use of Tablet- PCs the users never used the keyboard, maintaining the screen folded to the keyboard all the time while holding them with the forearm and using the pencil with the other hand. After some 15 minutes most users started to feel the weight of the device, and then they looked for different supporting points to hold it (on their legs while sitting or on a chair, *etc.*). Initially many users had problems with the use of gestures to edit their sketches and freehand writing texts, especially when deleting; they had less problems with the other editing gestures (selection, move, copy, rotate, resize) especially after the second session. The screen size of the Tablet-PCs was perceived as sufficient and comfortable to work in brainsketching processes on the map.

### 6.5. Discussion

During the workshops the three evaluators groups stated that the main value of the application was that it allowed keeping in a single interface various elements which help to generate ideas and create new proposals for urban design: first, it was possible to specify ideas by mean of sketches in an individual as well as in a collaborative way; second, it provides an interface allowing them to easily make an interpretation of the data they are working with; third, it provides a shared workspace where they can analyze the various design alternatives; fourth, it provides a workspace where it is possible to visually associate ideas to physical locations. In the same way, evaluators expressed that mobility was a key feature to accomplish their task, which they normally do behind a desk when not using such a tool.

During the workshop we also analyzed the benefits evaluators foresee by using the tool on a regular basis in the long term. In this regards, the evaluators expressed that they see benefits on the support for team work, which was not frequently done due to the practical limitations of their work and on the creation of a historical record of the proposed ideas, which can on the long term build a valuable asset.

## 7. Conclusions and Outlook

We think that the main contribution of our work has been to identify the benefits of using geo-referenced information in knowledge creation and proposing a mechanism to put this idea into practice. In our software application this is achieved by associating GPS and location information with geo-referenced data and information over a map for knowledge creation in urban planning scenarios where previous knowledge creation is needed for sense-making. The visualization technology of knowledge and the use of mobile devices as support for knowledge management is a new field, which has already generated applications for different scenarios such as engineering, education economy and health [40]. Our application supports the visualization of information in a free and extensible way while the users are working in mobile co-located scenarios. It also promotes the collaboration in mobile scenarios by making use of *ad-hoc* wireless networks, which helps to transform tacit into explicit knowledge, promoting the elicitation, transmission and sharing of information based on sketches by supporting the dynamic of the dialectic and the social reflex ion and interaction of individuals and groups.

The knowledge creation success model developed emphasizes the need for knowledge creation systems to include both types of knowledge (tacit and explicit) and linkages or pointers to people with knowledge expertise. A better understanding of the various characteristics of the tacit knowledge dimension, as detailed in the present study, will assist researchers and practitioners in the development of more sophisticated knowledge management systems that can adequately address knowledge users' needs for both codified knowledge and interaction with human sources of knowledge. As future work we envisage to explore supporting combined virtual co-located and face-to-face co-located scenarios, by using HTML5 technology, which will allow users to access to the system using a web browser. Preliminary work has shown that it is easy to extend the current application for this purpose. HTML5 will allow mobile phone owners to run this tool in their devices, since most web-browsers for mobile phones are HTML5 compatible. However this will raise a new requirement for the tool, since it has been successfully tested for its usability with normal screen sizes. The tool running over devices with a reduced screen should then be tested again in order to validate the sketches approach or develop a more suitable HCI paradigm for this scenario.

## Figures and Tables

**Table 1.** The table shows the frequency a certain level of the Likert scale was chosen by the evaluators for each association of functional mode and requirement. **R1**—*Spiral conversion of tacit and explicit knowledge*. **R2**—*Support divergent and convergent thinking*. **R3**—*Support individual, dyadic, and group brainstorming*. **R4**—*Support flexibility in the granularity of planning*. **R5**—*Usage of visual mechanism, such as sketching and brainsketching*. **FM1**—*Brainwriting & brainsketching of ideas*. **FM2**—*Relevant information selection*. **FM3**. *Visual representation of knowledge created*.

	**Scores**				
	**Poor**	**Deficient**	**Fair**	**Good**	**Very good**
**First experimental scenario**					
**Functionality**					
**FM1** associated to **R1**			1	1	2
**FM1** associated to **R2**				3	1
**FM1** associated to **R3**			1	2	1
**FM2** associated to **R5**			1	2	1
**FM3** associated to **R5**				3	1
**FM1, FM2,** and **FM3** associated to **R4**				2	2
**Usability**					
Comprehension (understanding the application)			1	1	2
Learning (how to use the application)			2	2	
Operability (effort controlling the urban design collaborative task)			1	2	1
**Second experimental scenario**					
**Functionality**					
**FM1** associated to **R1**		1	1	2	
**FM1** associated to **R2**			1	2	1
**FM1** associated to **R3**			2	2	
**FM2** associated to **R5**				2	2
**FM3** associated to **R5**		1		2	1
**FM1, FM2,** and **FM3** associated to **R4**				2	2
**Usability**					
Comprehension (understanding the application)				3	1
Learning (how to use the application)			1	2	1
Operability (effort controlling the urban design collaborative task)			2	2	
**Third experimental scenario**					
**Functionality**					
**FM1** associated to **R1**			2	3	1
**FM1** associated to **R2**			1	3	2
**FM1** associated to **R3**		1	1	2	2
**FM2** associated to **R5**			1	2	3
**FM3** associated to **R5**			1	3	2
**FM1, FM2,** and **FM3** associated to **R4**				2	2
**Usability**					
Comprehension (understanding the application)				3	3
Learning (how to use the application)			1	3	2
Operability (effort controlling the urban design collaborative task)			2	3	1
